# Management of Root Resorption Using Chemical Agents: A Review

**DOI:** 10.7508/iej.2016.01.001

**Published:** 2015-12-24

**Authors:** Zahed Mohammadi, Zafer C. Cehreli, Sousan Shalavi, Luciano Giardino, Flavio Palazzi, Saeed Asgary

**Affiliations:** a *Iranian Center for Endodontic Research, Research Institute of Dental Sciences, Shaheed Beheshti University of Medical Sciences, and Iranian National Elite Foundation, Tehran, Iran****; ***; b * Department of Pediatric Dentistry, Faculty of Dentistry, Hacettepe University, Ankara, Turkey****; ***; c *Private Practitioner, Hamedan, Iran****; ***; d * Department of Periodontology, Endodontology, Pharmacology and Microbiology, Dental School, University of Brescia, Italy****; ***; e * Department of Odontostomatological and Maxillofacial Sciences, Federico II University of Naples, Italy****; ***; f *Iranian Center for Endodontic Research, Research Institute of Dental Sciences, Shahid Beheshti University of Medical Sciences, Tehran, Iran*

**Keywords:** Alendronate, Calcitonin, Citric Acid, Emdogain, Fluoride, Osteoclast, Root Resorption, Tetracycline

## Abstract

Root resorption (RR) is defined as the loss of dental hard tissues because of clastic activity inside or outside of tooth the root. In the permanent dentition, RR is a pathologic event; if untreated, it might result in the premature loss of the affected tooth. Several hypotheses have been suggested as the mechanisms of root resorption such as absence of the remnants of Hertwig's epithelial root sheath (HERS) and the absence of some intrinsic factors in cementum and predentin such as amelogenin or osteoprotegerin (OPG). It seems that a barrier is formed by the less-calcified intermediate cementum or the cementodentin junction that prevents external RR. There are several chemical strategies to manage root resorption. The purpose of this paper was to review several chemical agents to manage RR such as tetracycline, sodium hypochlorite, acids (citric acid, phosphoric acid, ascorbic acid and hydrochloric acid), acetazolamide, calcitonin, alendronate, fluoride, Ledermix and Emdogain.

## Introduction

Root resorption (RR) is the loss of dental hard tissues as a result of clastic activities within the pulp or periodontium. It might occur as a physiologic or pathologic phenomenon. RR in the primary dentition is a normal physiologic process except when it occurs prematurely [1, 2]. The initiating factors involved in physiologic RR in the primary dentition are not completely understood, although the process appears to be regulated by cytokines and transcription factors that are similar to those involved in bone remodeling [1]. Unlike bone that undergoes continuous physiologic remodeling throughout life, RR of permanent teeth does not occur naturally and is invariably inflammatory in nature. Thus, RR in the permanent dentition is a pathologic event and if untreated, this might result in the premature loss of the affected teeth [1-4].

***Etiology of root resorption***

RR occurs in two phases: injury and stimulation. Injury is related to non-mineralized tissues covering the external surface of the root (pre-cementum) or internal surface of the root canal (predentin) [2, 5]. The injury is similar to several types of RR and may be mechanical following dental trauma, surgical procedures and excessive pressure of an impacted tooth or tumor. It may also occur following chemical irritation after tooth whitening using hydrogen peroxide. Denuded mineralized tissue is colonized by multinucleated cells, which initiate the resorptive process [5]. 

Without further instigation of the clastic cells, resorption is self-limiting. If the damaged surface is small, repair with cementum-like tissue will occur within 2 to 3 weeks. If the damaged root surface is large, bone cells attach to the root in competition with cementum-producing cells and ankylosis takes place [1, 5]. Continuation of the active resorption process is dependent on a common stimulation factor of the osteoclastic cells, either infection or pressure. Its origin is different for each type of RR. Therefore, the various types of RR should be identified according to the stimulation factors. When these stimulation factors are identified, it will be possible to reverse the process by removing the etiological factors [1, 5]. 

**Mechanisms of root resorption**

Several hypotheses have been suggested regarding the mechanisms of RR. According to a hypothesis, the remnants of Hertwig's epithelial root sheath (HERS) surround the tooth root, like a net impart, which is resistant to resorption and subsequent ankylosis [3, 6]. Hasegawa *et al. *[7] showed that the protective role of HERS had not been established. However, they revealed that HERS cells, by producing specific matrix proteins including osteopontin, ameloblastin and bone morphogenic proteins (BMPs) play an important role in cemental repair subsequent to resorption. Another hypothesis is the absence of some intrinsic factors in cementum and predentin such as amelogenin or osteoprotegerin (OPG) [a member of tumor necrosis factor (TNF) superfamily], which act as the inhibitors of resorptive cells. 

OPG is a decoy receptor by binding to the receptor activator of nuclear factor *κ*B ligand (RANKL). Binding to OPG reduces RANKL concentration and thereby inhibits its ability to bind to receptor activator of nucleic factor *κ*B (RANK) receptors on the surface of osteoclast precursors (circulating monocytes) and stimulate osteoclast production [8]. 

Another hypothesis regarding some forms of external RR is the barrier formed by the less highly calcified intermediate cementum or the cementodentin junction. The intermediate cementum is the innermost layer of cementum which creates a barrier between the dentinal tubules and the periodontal ligament. Under normal conditions, this barrier does not allow irritants such as bacterial by-products to pass from an infected pulp space to stimulate an inflammatory response in the adjacent periodontal ligament. If the intermediate cementum is lost or damaged, pro-inflammatory mediators may diffuse from an infected pulp space into the periodontal ligament, setting up an inflammatory response and subsequent external inflammatory RR [9].

**Materials used to manage root resorption**

**Tetracyclines**

Tetracyclines, including tetracycline-HCl, minocycline, demeclocycline and doxycycline, are a group of broad-spectrum antibiotics that are effective against a wide range of microorganisms [10]. Tetracyclines are bacteriostatic in nature; which may be advantageous because in the absence of bacterial cell lysis, antigenic by-products such as endotoxins are not released [10]. Tetracyclines also have many unique properties other than their antimicrobial effect such as the inhibition of mammalian collagenases, which prevents tissue breakdown and the inhibition of clastic cells which results in anti-resorptive activity [11].

Inflammatory diseases such as periodontitis include a pathological excess of tissue collagenases, which may be blocked by tetracyclines, leading to enhanced formation of collagen and bone formation [12].

Based on the hypothesis that microorganisms reach the apical area of the recently replanted tooth from the oral cavity (or from contaminated root surfaces during extra-oral time), and considering the inhibitory action of tetracyclines in preventing this route of bacterial contamination, Cvek *et al.* [13] developed a new protocol for topical treatment of the exposed root with doxycycline before replantation, aiming to locally eliminate microorganisms from the root surface of an avulsed tooth in order to decrease the frequency of inflammatory response. The authors showed that topical doxycycline significantly increased the chances of successful pulp revascularization. The beneficial effect of soaking a tooth in doxycycline, was recently confirmed by Yanpiset and Trope [14].

Bryson *et al*. [15] evaluated the effect of minocycline on healing of replanted dog teeth after extended (60 min) dry time. Results showed that the roots with and without minocycline treatment showed no significant differences between the remaining root mass or the percentage of favorably healed root surfaces and no benefit was found from the use of topically applied minocycline in the attenuation or prevention of external RR.

Terranova [16] showed that tetracyclines promote fibroblast and connective tissue attachment and enhance regeneration of lost periodontal attachment to pathologic processes. Ma and Sae-Lim [17] revealed that soaking the avulsed monkeys’ teeth in minocycline after 60 min dry time, increased normal periodontal healing. However, there was no decrease in osseous replacement in the experimental teeth. 

**Sodium hypochlorite**

Because of its excellent antibacterial activity and its ability to dissolve necrotic tissues, various concentrations of sodium hypochlorite (NaOCl) have been recommended for removing necrotic periodontal ligament remnants [18]. The best results have been achieved with lower concentrations and consisted predominantly of long-term replacement resorption/ankylosis and short-term absence of resorption [15]. According to Sottovia *et al.* [19] protocols including application of 1% sodium hypochlorite in delayed replanted teeth, was ineffective in controlling RR.

**Acids**

***Hydrochloric acid***

Hydrochloric acid has also been used in association with an enzyme, hyaluronidase, with the aim of decalcifying the cementum without denaturing the collagen matrix and has been shown to significantly decrease RR [20]. Nordenram *et al.* [21] evaluated the use of hydrochloric acid alone but did not have favorable results. 

***Phosphoric acid***

Phosphoric acid at 50% concentration has been used for the same purpose, and the results revealed that its use alone increased the occurrence of RR [22]. According to Sottovia *et al.* [19] protocols including application of phosphoric acid in delayed replanted teeth, was ineffective in controlling RR.

***Citric acid***

In an attempt to expose collagen fibers on root cementum and promote a contact surface for re-attachment of periodontal ligament collagen fibers, treatment of root surface with citric acid has been proposed. Large number of ankylotic areas and replacement resorption has been observed after treating the root surface with citric acid [23-25]. After demineralizing the root surface with citric acid, Ripamonti and Petit [26], used a concentrate of allogenic fibronectin-fibrin with the aim of preventing ankylosis but did not succeed. 

***Ascorbic acid***

Ascorbic acid (vitamin C) is a fundamental vitamin in all body tissues. It is essential for the hydroxylation of proline and lysine, which are very important during the synthesis of collagen. Its absorption depends on tissue concentration. If available at an appropriate concentration, ascorbic acid is responsible for maintaining the efficacy and the phagocytosis activity of leucocytes [27].

Vitamin C also plays a role in osteogenesis by the activation of alkaline phosphatase and increase in the functional activity of osteoblasts [27]. Vitamin C is an acidic molecule and has properties capable of influencing tissue repair, which might play an important role in delayed tooth replantation [28, 29]. 

**Acetazolamide**

Acetazolamide is a carbonic anhydrase inhibitor that is used to treat several diseases such as glaucoma, epileptic seizures, cystinuria, periodic paralysis and dural ectasia [30]. Mori and Garcia [31] assessed the effectiveness of Acetazolamide solution as an intra-canal medicament to manage RR in delayed replanted teeth. Results revealed that after 60 days, Acetazolamide inhibited RR completely. 

**Calcitonin**

Calcitonin is a hormone synthesized by the thyroid gland which is a proven potent inhibitor of clastic cells and has been indicated for the treatment of external RR. The influence of this hormone as an intracanal dressing after tooth replantation has been investigated, and it has been observed that it causes a decrease in inflammatory RR and better control of the sequelae of dental trauma even in cases with uncertain prognosis [32].

In an experimental study in dogs, Caldart [33] histometrically evaluated delayed replantation in teeth with canals obturated with gutta-percha and zinc-oxide eugenol-based sealer or filled with the following medications: calcium hydroxide (CH) paste, calcitonin, and CH/calcitonin paste. After 30 days, definitive obturation was performed with gutta-percha. The CH/calcitonin paste was more effective in controlling inflammatory RR compared to the use of these medications alone. Root replacement resorption was more effectively controlled by the use of calcitonin, while the other medications had a similar effectiveness in controlling this event.

The association of calcitonin and CH as an intracanal medication for replanted teeth has been advocated mainly because of the recognized capacity of reducing the osteoclastic activity, interfering in the proliferation, motility, and vitality of these cells, and reducing the resorption rate. However, this association has not provided better results than CH alone [18].

**Alendronate**

Alendronate is currently being used to inhibit pathologic osteoclast-mediated hard tissue resorption in diseases such as Paget's disease, osteoporosis and osteoclastic malignancies of bone [34]. The affinity of alendronate for calcium phosphate and its tenacious binding to hydroxyapatite lead to its rapid uptake into the skeleton [35]. Once incorporated into the skeleton, the terminal half-life of alkaline phosphatase (ALN) activity has been determined to be as high as 1000 days in dogs [36]. The mechanism of osteoclast inhibition has been attributed to a decrease in osteoclast activity with minimal effects on recruitment [37], the interference of receptors on the osteoclasts for specific bone matrix proteins, promoting the production of an osteoclast-inhibitor by osteoclasts which reduces the life span and/or the number of differentiated osteoclasts, and the obstruction of resorption by interfering with the ruffled border of the osteoclast [38]. 

Levin *et al*. [39] assessed the effect of alendronate on RR of dried replanted dogs’ teeth and found that the alendronate-soaked roots had significantly more healing than the roots soaked in HBSS without alendronate. Furthermore, soaking in alendronate resulted in significantly less root mass resorption compared to teeth soaked in HBSS without alendronate. In another study, Kum *et al. *[40] indicated that alendronate had inhibitory effects on bacteria-stimulated osteoclast formation *in vitro*. Moreira *et al. *[41] showed that alendronate paste in polyethylene glycol was highly cytotoxic *in vitro *as well as *in vivo*. Lustosa-Pereira *et al. *[42] indicated that sodium alendronate was able to reduce the incidence of radicular resorption, but did not reduce dental ankylosis. Mori *et al.* [43] assessed the effect of a solution of alendronate as an intracanal therapeutic agent in rat teeth submitted to late re-implantation, morphometrically and microscopically. Findings revealed that the solution of alendronate and the CH paste limited the RR, but did not impair its occurrence. Komatsu *et al. *[44] evaluated the long-term inhibitory effects of topical alendronate in the rat replanted teeth. Findings showed that the inhibitory effects of topical alendronate were retained on root and bone resorption, but not on ankylosis and pulp mineralization, after 4 months. In a study on feline teeth, Mohn *et al.* [45] showed that, at a dose of 9 mg/kg twice weekly, alendronate effectively slowed or arrested the progression of resorption.

**Ledermix**

Ledermix is a glucocorticosteroid-antibiotic compound. Today, Ledermix paste remains a combination of the same tetracycline antibiotic, demeclocycline HCl (at a concentration of 3.2%), and a corticosteroid (1% triamcinolone acetonide), in a polyethylene glycol base [46]. The two therapeutic components of Ledermix (*i.e*. triamcinolone and demeclocycline) are capable of diffusing through dentinal tubules and cementum to reach the periodontal and periapical tissues [12]. Abbott *et al*. [47] showed that dentinal tubules were the major supply route of the active components to the periradicular tissues, while the apical foramen was not as significant as a supply route. Various factors can affect the supply of the active components to the periradicular tissues that include the presence or absence of the smear layer, cementum and other materials within the canal such as CH.

It has been demonstrated histologically that Ledermix eliminated experimentally induced external inflammatory RR *in vivo* [48]. Furthermore, it has been revealed that Ledermix paste had no damaging effects upon the periodontal membrane and that this paste was an effective medication for the treatment of progressive RR in traumatically injured teeth [48]. Ledermix inhibits mitosis of mouse fibroblasts while present in the concentrations range of 10^-^^3^ to 10^-^^6^ mg/mL reversibly. Furthermore, mixing with Pulpdent paste did not modify this anti-mitotic effect [49]. Thong *et al*. [50] found that inflammation of the periodontal ligament and inflammatory RR were markedly inhibited by Ledermix relative to untreated controls. Wong and Sae-Lim [51] evaluated the effect of immediate intracanal Ledermix on RR of delayed-replanted monkey teeth. For the experimental group, intracanal Ledermix was placed prior to extraction and replantation after 1-h bench dry. The positive control group was root filled and replanted after 1 h while the negative control group was root filled and replanted immediately. The negative control group produced highly significant favorable healing and unfavorable healing as compared to the Ledermix group. The Ledermix group only showed significantly higher occurrence of complete healing (35.46%) compared to the positive control group (16.58%) but there were no significant differences in the inflammatory root resorption and replacement resorption. Nevertheless, when the latter two unfavorable healing patterns were combined, there was a significantly lower overall unfavorable healing in the Ledermix group (64.54%) when compared to the positive control group (83.43%). This unfavorable healing outcome in the Ledermix group, however, was not significantly different from the favorable healing outcome with the same treatment. Bryson *et al*. [15] evaluated the effect of immediate intracanal placement of Ledermix paste on healing of replanted dog teeth after extended dry times (60 min). Their finding showed that the Ledermix-treated roots had significantly more healing and less resorption than the roots treated with CH. Root filling with Ledermix paste also resulted in significantly less resorption compared to those roots filled with CH. Chen *et al. *[52] evaluated the individual influence of triamcinolone and demeclocycline on external RR after extended extra-oral dry time (60 min). Their findings showed that the groups treated with Ledermix, triamcinolone and demeclocycline had significantly more favorable healing than the group filled with gutta-percha that were replanted after 60 min dry time (positive control) .There was no statistically significant difference between Ledermix group and triamcinolone group, while the tetracycline group showed less favorable healing than the negative control, Ledermix and triamcinolone groups. They concluded that corticosteroid and tetracycline, as anti-inflammatory and anti-resorptive agents, shut down or minimized the inflammatory reaction including clastic-cells mediated resorption, and thus promoted more favorable healing than the positive control group, which had no intra-canal medicaments. Furthermore, they forecasted that in severe traumatic injuries, where a large surface area of periodontal inflammation is expected, removing the pulp and placing corticosteroids into the canal at the emergency visit would become a standard protocol [52]. 

**Emdogain**

Enamel matrix derivative (EMD) in the form of a purified acid extract of proteins from pig enamel matrix (Emdogain; Straumann AG, Basel, Switzerland) has been successfully employed to restore functional periodontal ligament, cementum and alveolar bone in patients with severe attachment loss [53, 54].

One attractive possibility for application of Emdogain is for their use in replantation procedures with avulsed teeth. The underpinning mechanism is that root surface conditioning with amelogenin could prevent RR and ankylosis, and stimulate periodontal ligament formation after repositioning of the avulsed tooth. Some early case-reports and animal experimental findings suggested that EMD could be used as a bioactive root conditioning for reintegration of avulsed teeth [55], but subsequent studies have struggled with confirming this effect. A study in dogs showed no significant effects from EMD treatment on RR and ankylosis after 6 months [56]. In this study, the teeth were extracted and re-implanted after killing all cementoblasts on the root surface by drying the teeth in air for 60 min, before repositioning them. Accordingly, a clinical study using EMD to treat both previously ankylosed teeth reported that all teeth in the study showed clinical signs of ankylosis and concluded that EMD alone was not sufficient to cure or prevent ankylosis [57]. Another clinical study, however, assessed the clinical outcome of 22 avulsed permanent incisors replanted with EMD and showed significantly less inflammation and RR in treated teeth compared with a historical control group from the same region [58]. Also, a study using a protocol for replantation of avulsed teeth called anti-resorptive regenerative therapy (ART) that includes local application of glucocorticoids and EMD prior to the replantation procedure, reported that when ART was used on avulsed teeth that had been stored non-physiologically for longer periods (typically hours), three of eight teeth healed with a functional periodontal ligament [59]. It thus seems that when used together with anti-inflammatory drugs, EMD can support functional healing and periodontal regeneration after replantation, even when the avulsed teeth have a severely compromised cementum layer.

In summary, interesting data on the utilization of amelogenin for replantation of avulsed teeth has accumulated. It seems plausible that amelogenin can stimulate regeneration of the tooth attachment apparatus even in cases where the tooth has been stored for significant time outside the oral cavity. However, additional treatment is needed to ensure stable and predictive treatment outcomes, and new protocols for combination of amelogenin treatment with anti-inflammatory and anti-microbial drugs must be developed and tested before the full potential of amelogenin can be exploited in dental traumatology.

**Fluoride**

Several studies have recommended the use of fluoride solutions in different forms and concentrations to treat the root surface in cases of delayed tooth replantation, assuming that the demineralized dentin surface would be more prone to fluoride incorporation and might become more resistant to resorption. 

Among the fluoride solutions, the use of 2% acidulated sodium phosphate fluoride has shown a decreased inflammatory RR and the predominance of areas of ankylosis and replacement resorption [60-62]. According to Bjorvatn *et al.* [63] fluoride probably acts directly on the bone tissue, cementum and dentin, by converting hydroxyapatite into fluorapatite, or by a specific inhibitory action on the clastic cells, or even an association of both hypotheses. Ekstrand *et al. *[64] showed that fluoride was able to inhibit microbial growth and metabolism and therefore decreasing cell pH. Barbakow *et al*. [65] did not find any difference between repairing ability of acidulated sodium fluoride and neutral sodium fluoride. 

In a study on monkeys' teeth, Barbakow *et al.* [65] showed that pre-treatment of the tooth roots with 2% acidulated sodium fluoride did not reduce RR and ankylosis. Bjorvatn *et al.* [63] indicated that application of SnF_2_ to the root surface prior to replantation effectively reduces resorptive processes during the first postoperative weeks. By subsequently treating the root surface with tetracycline, the adverse effect of SnF_2_ on periodontal connective tissue repair reduced. In a study on dogs' teeth, Selvig *et al.* [66] showed that root surface treatment with SnF_2_ followed by tetracycline resulted in complete absence of inflammatory resorption and ankylosis in the short-term experiment. In another study, Selvig *et al.* [67] indicated that reducing the strength of the SnF_2_ solution from 1% to 0.1% may result in less persistent inflammation, however at the cost of less complete prevention of inflammatory resorption and ankylosis. Kameyama *et al. *[68] revealed that the administration of fluoride suppressed RR induced by mechanical injuries of the periodontal soft tissues.

Wikesjo *et al.* [69] evaluated healing, with emphasis on RR, following root surface treatment with 1% aqueous SnF_2_, saturated citric acid, or saline control in conjunction with periodontal flap surgery. According to their findings SnF_2_-treated teeth healed with significantly longer junctional epithelium, less connective tissue repair to the root surface, and less bone regeneration than citric acid and control teeth. New cementum formation was limited in all treatment groups. RR was observed in almost all teeth exhibiting connective tissue repair, however to a lesser amount and not as frequent in SnF_2_ treated teeth due to limited connective tissue repair. Poi *et al. *[70] showed that 2% acidulated-phosphate sodium fluoride was not able to prevent RR in delayed tooth replantation in rats. Gulinelli *et al.* [71] assessed the influence of 15% propolis and the fluoride solution used as root surface treatment on the healing process after delayed tooth replantation. Findings revealed that there was similar external root resorption in propolis and fluoride groups.

## Conclusion

*i)* Tetracyclines have antimicrobial and anti-resorptive activity.

*ii)* Treatment of root surface with SnF_2_ followed by tetracycline resulted in complete absence of inflammatory resorption and ankylosis in short-term. 

*iii)* Ascorbic acid is capable of influencing tissue repair. 

*iv)* Acetazolamide is effective against root resorption. 

*v)* Combining calcium hydroxide with calcitonin seems to be effective in controlling inflammatory root resorption. 

*vi)* Alendronate is effective on inflammatory root resorption but has no effect on replacement resorption. 

*vii)* Ledermix prevents inflammatory root resorption.

*viii)* Emdogain can support functional healing and periodontal regeneration after replantation, even when the avulsed teeth have a severely compromised cementum layer.
